# Prospective longitudinal analysis of antibody response after standard and booster doses of SARS-COV2 vaccination in patients with early breast cancer

**DOI:** 10.3389/fimmu.2022.1028102

**Published:** 2022-11-17

**Authors:** Jinyong Kim, Jiyun Jeong, Chan Mi Lee, Dae-Won Lee, Chang Kyung Kang, Pyeong Gyun Choe, Nam Joong Kim, Myoung-don Oh, Chang-Han Lee, Wan Beom Park, Kyung-Hun Lee, Seock-Ah Im

**Affiliations:** ^1^ Department of Internal Medicine, Seoul National University College of Medicine, Seoul, South Korea; ^2^ Department of Biomedical Sciences, Seoul National University College of Medicine, Seoul, South Korea; ^3^ BK21 FOUR Biomedical Science Project, Seoul National University College of Medicine, Seoul, South Korea; ^4^ Wide River Institute of Immunology, Seoul National University, Hongcheon, South Korea; ^5^ Department of Pharmacology, Seoul National University College of Medicine, Seoul, South Korea; ^6^ Cancer Research Institute, Seoul National University, Seoul, South Korea

**Keywords:** SARS-CoV-2, COVID-19, breast cancer, neutralizing antibodies, serological response, adjuvant chemotherapy

## Abstract

**Introduction:**

Severe acute respiratory syndrome coronavirus 2 (SARS-CoV-2) and its variants brought waves of pandemics with breakthrough infections in vaccinated individuals. We analyzed the antibody responses after primary and booster vaccination in healthy controls (HC) and patients with early breast cancer (BC).

**Methods:**

In this prospective longitudinal cohort study, the binding activity of serum antibody level against spike proteins and antigens of SARS-CoV-2 variants was measured within 21 days after each vaccination in the BC group and HC group.

**Results:**

All participants, 40 in the BC and 20 in the HC group, had increased antibody response after vaccination. BC group, however, had weaker humoral responses than the HC group (IgG: 1.5, 2.3, 2.5-folds in BC vs. 1.9, 3.6, 4.0-folds in HC after each dose; IgA: 2.1, 3.0, 3.6-folds in BC vs. 4.2, 10.4, 5.2-folds in HC after each dose, respectively). Those under concurrent cytotoxic chemotherapy had weaker antibody response than the non-cytotoxic treatment group and HC. Adjunct use of steroids and age were not significant risk factors. The levels of binding antibody against the Delta and the Omicron (BA1) variants were lower than the wild-type, especially in BC.

**Conclusion:**

In the waves of new sub-variants, our study suggests that an additional dose of vaccinations should be recommended according to the anti-cancer treatment modality in patients with BC who had received booster vaccination.

## Introduction

Since the outbreak of coronavirus disease-19 (COVID-19) in December 2019, waves of infection by severe acute respiratory syndrome coronavirus 2 (SARS-CoV-2) variants of concern (VOCs) have affected over 590 million cumulative cases worldwide as of 22 August 2022 (1, 2). Last year, the Delta (B.1.617.2 and AY lineages) variant had dominated globally with a high risk of hospitalization and mortality (3–6), until Omicron (B.1.1.529, BA.1, BA.1.1, BA.2, BA.3, BA.4 and BA.5 lineages) variant became prevalent with greater replication rate and evasion of humoral immunity (7).

Initial studies indicated that standard doses of vaccines against COVID-19, including the BNT162b2 vaccine (Pfizer–BioNTech (PZ)), mRNA-1273 vaccine (Moderna), ChAdOx1 nCoV-19 (Oxford-AstraZeneca (AZ)), and Ad26.COV2.S vaccine (Janssen) provided effective protection against symptomatic diseases in the first wave (8–11). Later, while vaccination had marginally decreased efficacy against the Delta variant, it offered only limited protection against Omicron variants (12, 13). Subsequent studies reported that additional booster dose substantially increased vaccine efficacy against Omicron variants (13).

The necessity of booster doses had been prioritized by most authorized global guidelines in more vulnerable populations, including patients with cancer (14–19). A previous study showed that breast cancer was the second most common type of cancer among cancer patients with COVID-19 infection, following hematological malignancies (20). Another study showed that the patients with breast cancer infected with COVID-19 disease showed 10.9% of all-cause in-hospital mortality rate, and 15-30% of long-term COVID-19 sequelae incidence rate (21). Therefore, obtaining antiviral immunity through vaccination is very important for patients with breast cancer. However, patients with breast cancer receive various modalities of medical treatments, including hormonal therapy, molecular targeted therapy, and chemotherapy, often with corticosteroids that could interfere with immunity (22), and the efficacy of COVID-19 vaccination and boosters on the patients under different anti-cancer treatments has not been clearly evaluated yet.

In this study, we aimed to assess the antigenicity of the primary and booster doses of COVID-19 vaccines against SARS-CoV-2 VOCs in patients with early breast cancer compared to healthy controls.

## Materials and methods

### Study design and participants

Our study design was a prospective longitudinal cohort study. Patients with early breast cancer who were 20 years or older and received systemic anti-cancer treatments for breast cancer at Seoul National University Hospital (SNUH) were recruited as in previous study (23). Controls were healthcare workers at SNUH who did not have any systemic diseases. Participants with confirmed COVID-19 infections were excluded. The Institutional Review Board approved the study (IRB No. 2103-121-1206 and 2102-032-1193). Written informed consents were obtained from all participants before the initial vaccination. All the data from the patients were anonymized and de-identified prior to analysis.

Definition of early breast cancer was confined to breast disease with or without the involvement of regional lymph nodes but without distant metastasis. Medical anti-cancer treatments comprised cytotoxic chemotherapy, molecular targeted therapy, and endocrinal treatment. The patients were stratified by the treatments they received within 28 days of vaccination: cytotoxic chemotherapy group, non-cytotoxic anticancer group, and post-treatment group. The cytotoxic drugs included anthracyclines, cyclophosphamide, docetaxel, paclitaxel, and carboplatin. Non-cytotoxic treatments were molecular targeted therapy and endocrinal therapy. Targeted antibodies were trastuzumab and pertuzumab. Endocrinal therapy was chosen among tamoxifen, letrozole, anastrozole, and gonadotropin-releasing hormone agonists in the context of the patient’s menopausal status and clinical conditions. Post-treatment group did not receive any anti-cancer treatments within 28 days of vaccination. Corticosteroid doses equivalent to or more than 10 mg of prednisolone within 14 days of vaccination were analyzed for immune-modulatory effect.

Primary vaccinations were defined as either two doses of PZ, Moderna, AZ, or a single dose of Janssen. A booster injection was defined as the additional dose of PZ or Moderna to primary vaccinations. Cross-injection among different vaccines was allowed. The patients who received a single dose of the Janssen vaccine and booster dose were only included in the first dose and the booster dose group for analysis. The serum of participants was collected at baseline before the vaccination, three weeks after a first and second dose of primary vaccinations, before the booster dose, and three weeks after the booster dose.

Our primary endpoint was the level of antibody response of the COVID-19 vaccinations and boosters in patients with early breast cancer compared to healthy controls. Secondary outcomes were the effect of age and corticosteroid use on the immunogenicity of vaccines and the efficacy against variants of COVID-19. Blood samples were collected at baseline and within 21 to 28 days after the vaccinations and booster injections.

### Preparation of recombinant SARS-CoV-2 antigens

pcDNA3.1 SARS-CoV-2 S D614G was a gift from Jeremy Luban (Addgene plasmid # 158075; http://n2t.net/addgene:158075; RRID: Addgene_158075). SARS-CoV-2 S D614G protein, RBD_wt_, RBD_δ_, and RBD_ο_ were produced in Expi293 cells (Thermo Fisher Scientific) and were purified using Ni-NTA agarose resin (Thermo Fisher Scientific) affinity chromatography, as described previously (24, 25).

Briefly, Expi293 cells were cultured at 37 °C with 5% CO_2_ for five days after transfection of each plasmid encoding SARS-CoV-2 S D614G protein, RBD_wt_, RBD_δ_, or RBD_ο_ (BA1). The supernatant was collected and passed over the Ni-NTA agarose resin column three times. After washing with 100 mL of phosphate-buffered saline (PBS), the his-tagged protein was eluted by elution buffer (pH8.0, 50 mM sodium phosphate, 300 mM NaCl, and 250 mM imidazole). Finally, samples were buffer-exchanged into pH 7.4 PBS using Amicon Ultra-4 (Merck Millipore, Burlington, MA, USA) spin columns with a 10 kDa cutoff. The purity of purified samples was assessed by 14% SDS-PAGE gel.

### Binding antibody ELISA

The binding activity of serum antibody (Ab) level against each SARS-CoV2 antigen (spike protein, RBD_wt_, RBD_δ_, RBD_ο_, and nucleocapsid) was measured by enzyme-linked immunosorbent assay (ELISA), as described previously (24–26). The 96-well polystyrene ELISA plate (Thermo Fisher Scientific) was coated with 100 ng of each antigen per well overnight at 4 °C. Each well was blocked with 100 μl of PBS (pH 7.4) containing 3% bovine serum albumin (BSA) for an hour at room temperature, and then the plate was washed four times with the PBST buffer (PBS with 0.05% Tween 20). The diluted serum samples (1:10, 1:50, 1:250) were added into wells, and each sample was diluted five-fold serially. After incubating at room temperature for an hour, wells were washed with PBST four times. Then, goat anti-human IgG Fc Ab-conjugated with HRP (1:12,000, Arigobio, Hsinchum Taiwan) was added and incubated at room temperature for an hour. Alternatively, mouse anti-human IgA (1:100) Ab was added and incubated at room temperature for an hour, and then anti-mouse IgG (H+L)-conjugated with HRP (Thermo Fisher Scientific) was added and incubated at room temperature for an hour for IgA detection. After washing with the PBST four times, 50 μl of 3,3’,5,5’-tetramethylbenzidine was added per well as chromogen substrate. The plate was kept at room temperature for 20 min, and the reaction was terminated by adding 50 μl of 2M H_2_SO_4_. Finally, absorbance was measured at 450nm with an Infinite 200 PRO-Nano Quant microplate reader (Tecan Trading AG, Mannedorf, Switzerland). Relative binding activity was calculated as the ratio of the binding activity at a certain time point to the binding activity at pre-vaccination in the same donor.

### Statistical analyses

Data are presented as mean ± standard deviation of the mean (s.d.) and as dot plots. Multiple unpaired T-test was performed to compare levels of immune responses between two groups. *P* < 0.05 was considered indicative of statistical significance. All statistical analyses were two-tailed and used GraphPad Prism 9 (GraphPad Software, La Jolla, CA, USA). All graphs were generated using GraphPad Prism 9.

## Results

### Study participants

Initially, 41 patients with early breast cancer and 20 healthy controls were recruited. One participant with breast cancer was excluded due to confirmed COVID-19 infection prior to the study. All breast cancer patients were female, and the median age was 51.5 years. During the primary vaccinations, PZ vaccines were most commonly injected in 32 (80.0%), followed by Moderna (4 of 40, 10.0%), AZ (3 of 40, 7.5%), and Janssen (1 of 40, 2.5%). Three-quarters of patients received anti-cancer treatments within 28 days of initial vaccinations. Of 15 patients who received chemotherapy regimens with cytotoxic drugs, two patients received concurrent HER2 targeted treatment. The non-cytotoxic treatment group comprised 15 patients who received hormonal therapies. Post-treatment group comprised 10 patients who were not under active anti-cancer treatments. Corticosteroids were only used in adjunct to cytotoxic chemotherapy.

Later at the time of receiving booster doses, however, all patients had completed cytotoxic chemotherapy and were re-categorized into a non-cytotoxic group or post-treatment group. Most patients (28 of 40, 70.0%) were on endocrinal treatments or targeted therapies, while 12 patients (30.0%) did not have further anti-cancer treatments. All except three patients received booster vaccination with either PZ (33 of 40, 82.5%) or Moderna (4 of 40, 10.0%). One patient in cytotoxic group had symptomatic COVID-19 infection after the second vaccination and did not receive booster dose. The other two patients, also in cytotoxic group, refused booster doses. In the healthy control group, all were female, and the median age was 31.0 years. All participants received PZ vaccines The patient characteristics are shown in **Table 1**.

**Table 1:** Detailed clinical information of patients.

	All	Cytotoxic chemotherapy	Non-cytotoxic treatment	Post-treatment	Control
Total number	40	15 (37.5%)	15 (37.5%)	10 (25.0%)	20
Sex (Female)	40 (100.0%)	15 (100.0%)	15 (100.0%)	10 (100.0%)	20 (100.0%)
Age (median, range)	51.5 (25-46)	50.0 (40-65)	49.0 (35-63)	52.0 (38-58)	31.0 (25-61)
Primary vaccine					
PZ	32 (80.0%)	10 (66.7%)	14 (93.3%)	8 (80.0%)	20 (100.0%)
Moderna	4 (10.0%)	3 (20.0%)	1 (6.7%)	0 (0.0%)	0 (0.0%)
Janssen	1 (2.5%)	1 (6.7%)	0 (0.0%)	0 (0.0%)	0 (0.0%)
AZ	3 (7.5%)	1 (6.7%)	0 (0.0%)	2 (20.0%)	0 (0.0%)
Booster vaccine					
PZ	33 (82.5%)	9 (60.0%)	14 (93.3%)	10 (100.0%)	20 (100.0%)
Moderna	4 (10.0%)	3 (20.0%)	1 (6.7%)	0 (0.0%)	0 (0.0%)
No booster	3 (7.5%)	3 (20.0%)	0 (0.0%)	0 (0.0%)	0 (0.0%)
Anticancer treatments <28 days before the vaccination	30 (75.0%)	15 (100.0%)	15 (100.0%)	0 (0.0%)	–
Chemotherapy only	13 (32.5%)	13 (86.7%)	0 (0.0%)	0 (0.0%)	–
Chemotherapy + Targeted therapy	2 (5.0%)	2 (13.3%)	0 (0.0%)	0 (0.0%)	–
Endocrinal therapy	15 (37.5%)	0 (0.0%)	15 (100.0%)	0 (0.0%)	–
Post-treatment	10 (25.0%)	0 (0.0%)	0 (0.0%)	10 (100.0%)	–
No anticancer treatment	–	–	–	–	20 (100.0%)
Anticancer treatment <28 days before the booster	26 (70.3%)	11 (29.7%)	15 (100.0%)	0 (0.0%)	–
Endocrinal therapy	24 (63.9%)	9 (81.8%)	15 (100.0%)	0 (0.0%)	–
Endocrinal therapy + targeted therapy	1 (2.7%)	1 (9.1)	0 (0.0%)	0 (0.0%)	–
Targeted therapy	1 (2.7%)	1 (9.1)	0 (0.0%)	0 (0.0%)	–
Post-treatment	11 (29.7%)	1 (9.1%)	0 (0.0%)	10 (100.0%)	–
No anticancer treatment	–	–	–	–	20 (100.0%)
Corticosteroids (≥10 mg prednisolone equivalent) <14 days before the vaccination	8 (20.0%)	8 (53.3%)	0 (0.0%)	0 (0.0%)	0 (0.0%)

PZ, Pfizer; AZ, AstraZeneca.

**Figure 1:**
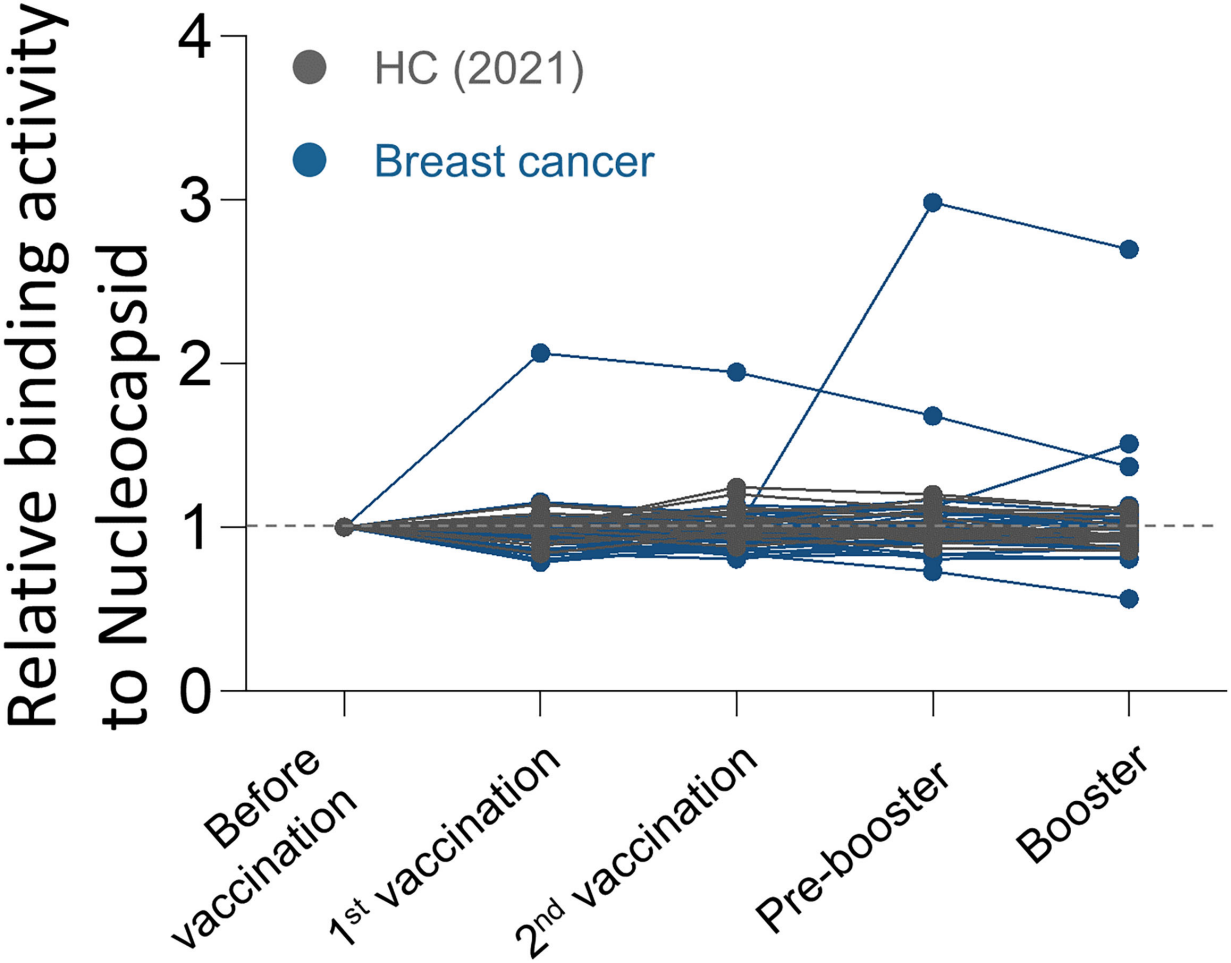
Antibody responses to SARS-CoV-2 nucleocapsid protein over time in breast cancer patients. Binding activities of IgG to SARS-CoV-2 nucleocapsid protein were determined using anti-IgG antibodies.

Prior to analysis, the SARS-CoV-2 nucleocapsid (NC) protein reactivity was examined to detect the participants who experienced asymptomatic COVID-19 infections. As a result, two additional breast cancer patients with NC-positivity were excluded from the study (**Figure 1**).

### Humoral immune responses in breast cancer patients

**Figure 2:**
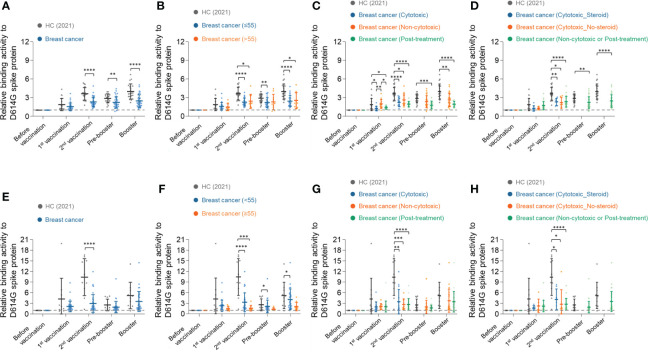
Longitudinal humoral immune responses to SARS-CoV-2 spike D614G protein in breast cancer patients. **(A)** Serum IgG binding activities to SARS-CoV-2 spike D614G in healthy control group and breast cancer patients group were determined using anti-IgG antibodies. The diluted serum samples (1:10) were incubated with SARS-CoV-2 spike D614G protein and then detected by streptavidin-HRP. **(B–D)** Breast cancer patients were stratified by their age **(B)**, types of anti-cancer therapy **(C)**, and use of adjunct steroids **(D)**. **(E–H)** Serum IgA binding activities to SARS-CoV-2 spike D614G in the healthy control group and breast cancer patients group were determined using anti-IgA antibodies. **(F–H)** Breast cancer patients were stratified by their age **(F)**, types of anti-cancer therapy **(G)**, and use of adjunct steroids **(H)**. Statistical analyses were performed using the Multiple unpaired T-test in GraphPad Prism (*P < 0.05, **P < 0.01, ***P < 0.001, ****P <0.0001).

The binding activity of serum IgG against recombinant D614G spike protein (**Supplementary Figure 1**) was examined in the serially diluted serum samples by enzyme-linked immunosorbent assay (ELISA) (**Supplementary Figure 2**). For more detailed analysis, the ELISA results of 10-fold diluted serum samples were used. Consistent with the previous report (24, 26), the healthy control group showed 1.9-fold, 3.6-fold, and 4.0-fold increase in IgG response against SARS-CoV-2 spike D614G compared to the baseline after the first, second, and booster doses of vaccinations, respectively (**Figure 2A**). On the other hand, the breast cancer group showed weaker humoral immune responses than the healthy control. The breast cancer group showed 1.5-fold, 2.3-fold, and 2.5-fold increased antibody response against SARS-CoV-2 spike D614G compared to the baseline after the first, second, and booster doses of vaccinations, respectively (**Figure 2A**).

Next, we stratified the breast cancer patients by their age, types of anti-cancer therapy, and use of adjunct steroids, and analyzed the antibody responses in each subgroup. In our study, most patients with breast cancer were under 65 years old. The patients were divided into an elderly group (>55 years old) and a younger group (≤55 years old), and the antibody response was equivalent in the two groups (**Figure 2B**). We also confirmed using Pearson’s correlation analysis that there was no significant correlation between age and humoral immune responses in the BC group **(** **Supplementary Figure 3**).

In the analysis of antibody response among patients treated with different anticancer therapies, the breast cancer patients who had concurrent chemotherapy showed significantly lower antibody responses against SARS-CoV-2 Spike D614G protein than the non-cytotoxic treatment group but equivalent to the post-treatment group after the first vaccination (**Figure 2C**). All groups showed increased binding activity after the second vaccination. At the time point of the pre-booster, all patients had completed cytotoxic chemotherapy and were classified into the non-cytotoxic or post-treatment group. The non-cytotoxic treatment group showed a higher antibody response than the post-treatment groups throughout the study (**Figure 2C**).

Next, we compared the antibody responses of breast cancer patients according to the use of adjunct steroids. Steroids were used only in the chemotherapy group as pretreatment to ameliorate hypersensitivity reaction and emesis. The antibody responses, however, did not show a significant difference with the use of adjunct steroid among patients who received cytotoxic treatment during the primary vaccinations (**Figure 2D**). No patients received cytotoxic chemotherapy and adjunct steroids during pre-booster and booster.

Similar to IgG responses, IgA responses against SARS-CoV-2 spike D614G in the healthy control group showed a higher fold increment than in the breast cancer group. The healthy control group showed a 4.2-fold, 10.4-fold, and 5.2-fold increase in IgA response against SARS-CoV-2 spike D614G compared to the baseline after the first, second, and booster doses of vaccinations, respectively (**Figure 2E**). In comparison, the breast cancer group showed 2.1-fold, 3.0-fold, and 3.6-fold increased antibody responses, respectively (**Figure 2E**). The younger group (≤55 years old) showed higher binding activities to SARS-CoV-2 spike D614G, but there was no statistical significance (**Figure 2F**). In addition, the types of anticancer therapies and adjunct steroids did not affect IgA response in BC patients (**Figures 2G, H**).

### Humoral immune responses against SARS-CoV-2 VOCs in breast cancer patients

**Figure 3:**
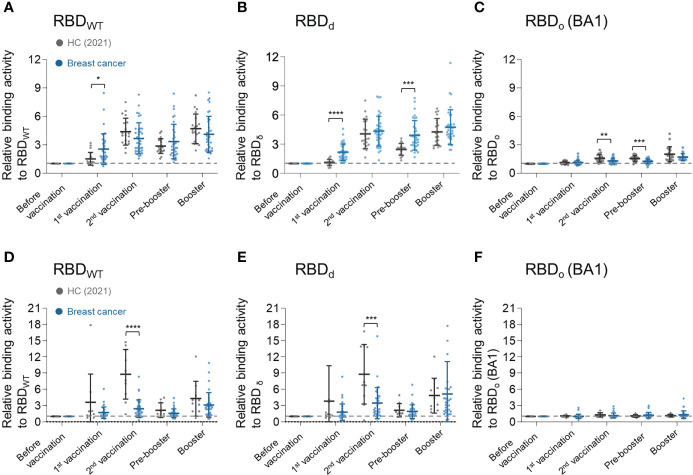
Longitudinal humoral immune response analysis to SARS-CoV-2 vaccine for SARS-CoV-2 VOCs. **(A–C)** Serum IgG binding activities against **(A)** RBD_wt_, **(B)** RBD_δ_, and **(C)** RBD_ο_. **(D–F)** Serum IgA binding activities against **(D)** RBD_wt_, **(E)** RBD_δ_, and **(F)** RBD_ο_. Binding activities of serum antibodies to RBD of SARS-CoV-2 variants were determined using anti-IgG or anti-IgA antibodies. In PBS, diluted plasma samples (1:100) were incubated with each biotinylated SARS-CoV-2 antigen and then detected by streptavidin-HRP. Statistical analyses were performed using the Multiple unpaired T-test in GraphPad Prism (*P < 0.05, **P < 0.01, ***P < 0.001, ****P <0.0001).

The binding activities of the serum antibodies produced by the vaccination to the SARS-CoV-2 VOCs were analyzed using recombinant RBD_wt_, RBD_δ,_ and RBD_ο_ proteins (**Supplementary Figure 1**). Serum IgG and IgA antibodies of breast cancer patients generally showed weaker binding activities for RBD_wt_ and RBD_δ_ than those of the healthy control group (**Figures 3A, B, D, E**). On the other hand, breast cancer patients had significantly weaker IgG binding activity to RBD_ο_ after the second vaccination than healthy control. After the booster shot, however, the IgG binding properties of breast cancer patients became equivalent to those of healthy control (**Figure 3C**). There was no statistical difference in IgA response between the healthy control group and breast cancer patients (**Figure 3F**).

## Discussion

In this study, we compared the antibody response of a homogeneous group of patients with early breast cancer under a variety of anti-cancer treatments with those of healthy controls. Our study highlighted that the patients with breast cancer are recommended for an additional booster dose, considering the gradually increasing but lower antibody response than the healthy controls after primary doses and one booster dose of vaccines.

As expected, patients with breast cancer showed lower antibody response than healthy control, but age was not a factor affecting the antibody response of breast cancer in our study (**Figures 2A, B, E, F**). It is well known that the immune response induced by vaccines weakens with age (27), and most significantly after 80 years old. It might be because all patients with early breast cancer who participated in this study were under the age of 65 and were in the acceptable range of similar levels of immunogenicity.

Types of concurrent anti-cancer treatment, however, significantly affected the humoral immune responses by vaccination in breast cancer patients. As reported in previous studies (28), breast cancer patients undergoing cytotoxic chemotherapy within 28 days of vaccinations had significantly lower anti-spike antibody levels. The use of steroids in adjunct to chemotherapy had only minimal effect on the antibody response of the breast cancer group despite known immunosuppressive properties (**Figure 2D**) (29), probably due to the overwhelming effect of cytotoxic chemotherapy.

Interestingly, after the first and second doses of vaccinations, patients on endocrine therapy or targeted therapy showed higher antibody responses than the cytotoxic and post-treatment group (**Figure 2C**) but a lower antibody level than healthy control. Previous studies have reported that male patients were more susceptible to severe COVID-19, suggesting estrogen’s protective role in controlling pro-inflammatory cytokines (30, 31). On the other hand, endocrinal therapies are thought to suppress the anti-inflammatory function of estrogen to improve the function of anti-tumor immune cells and reduce the number of immunosuppressive cells (32). The effect of endocrinal therapies, however, had been indeterminant despite multiple prospective studies, mainly due to the small number of patients receiving hormonal therapy (33, 34). The weighted risk of thrombosis by vaccination, in addition to tamoxifen, was also of great concern. In a cohort study that included people treated with tamoxifen, the thrombotic risk in people vaccinated with either AZ or PZ was primarily equivalent to that of general populations (35). Rather, the rates of pulmonary embolism were higher in those infected with COVID-19 compared to vaccinated populations. These enhanced humoral immune responses showed that the vaccinations could maximize the protection against severe COVID-19 disease in patients with estrogen suppression.

Notably, at the pre-booster time point, the binding activities of the HC group more rapidly declined but were still higher than those of the BC group. The patients with cancer were highly encouraged and were prioritized to receive a booster dose of vaccination. Considering that the median interval between the second and the booster dose was 3.5 months (range 1.9-5.7) in the BC group and 7.9months (range 7.2-8.4) in the HC group, it is reasonable that the HC group exhibited a more significant decline at pre-booster due to longer interval. Still, the HC group had higher binding activity than the BC group at pre-booster. Our data supported that booster doses might be helpful for patients with breast cancer.

The emergence of new variants of the SARS-CoV-2 virus resulted in breakthrough infections in previously infected or vaccinated individuals because the accumulation of mutations weakens binding activity against spike proteins (26). Such tendency was also consistently found in our data (**Figure 3**), with notably lower RBD binding activities of anti-spike antibodies in breast cancer patients than in the healthy control group.

The limitations of our study were intrinsic to a small number of participants, with only a few patients receiving targeted treatments. Nonetheless, we recruited a homogeneous group of patients with early breast cancer and were also able to evaluate the effect of various anti-cancer treatments, especially endocrinal therapy. The difference in vaccine types between breast cancer patients and the healthy control group may also interfere with data analyses. Recent data, however, showed that the booster dose with mRNA vaccines in Janssen-primed recipients showed sufficient immunogenicity (36). In our data, all patients who received the adenoviral vaccines had heterologous booster vaccines with mRNA vaccines, and the binding activities did not significant differ among vaccine types. This may partly be due to the small number of the patients, but our data indicated the equivalent binding activities with “mix and match” vaccination strategy.

## Conclusion

Currently, two new sub-variants of Omicron (BA.4 & BA.5) are spreading worldwide, raising concerns about their transmissibility amid the ongoing global Covid-19 pandemic. Our data showed that the patients with breast cancer have antibody responses lower than healthy controls that increase with booster doses, underscoring the necessity of additional booster vaccinations in breast cancer patients receiving anti-cancer chemotherapy.

## Data availability statement

The raw data supporting the conclusions of this article will be made available by the authors, without undue reservation.

## Ethics statement

The studies involving human participants were reviewed and approved by Institutional Review Board, Seoul National University Hospital. The patients/participants provided their written informed consent to participate in this study.

## Author contributions

K-HL and WBP conceptualized and designed the project. K-HL, WBP, C-HL, JK, JJ, and CML analyzed the data. K-HL WBP, JK, CML, D-WL, CKK, PGC, NJK, M-dO, and S-AI collected the human serum. C-HL and JJ performed serologic analysis. K-HL, WBP, C-HL, JK, and JJ wrote the manuscript with the help of all authors. K-HL and WBP had full access to all data in the study and took responsibility for the integrity of the data, as well as for the manuscript. All authors contributed to the article and approved the submitted version.

## Funding

This work was supported by the Bio & Medical Technology Development Program of the National Research Foundation (NRF) and was funded by the Korean government (MSIT) (grant No. 2021M3A9I2080498 to W. B. Park). This research was supported by the Ministry of Trade, Industry & Energy (MOTIE), Korea Institute for Advancement of Technology (KIAT) (Neutralizing Antibody Development Support Center, No. P0017147, to C.-H. Lee). This work was supported in part by the SNUH Research Fund (grant No. 04-2021-0250 to K.-H. Lee).

## Conflict of interest

The authors declare that the research was conducted in the absence of any commercial or financial relationships that could be construed as a potential conflict of interest.

## Publisher’s note

All claims expressed in this article are solely those of the authors and do not necessarily represent those of their affiliated organizations, or those of the publisher, the editors and the reviewers. Any product that may be evaluated in this article, or claim that may be made by its manufacturer, is not guaranteed or endorsed by the publisher.

## Supplementary material

Supplementary Figure 1 Purification of recombinant RBD proteins of SARS-CoV-2 variants and D614G spike protein. The purity of the purified recombinant protein was determined by non-reducing and reducing SDS-PAGE gels. M, protein ladder.

Supplementary Figure 2 Longitudinal humoral immune responses to SARS-CoV-2 spike D614G protein in breast cancer patients. The serially diluted serum IgG binding activities to SARS-CoV-2 spike D614G in the healthy control group and breast cancer patients group were determined using anti-IgG antibodies. The diluted serum samples were incubated with SARS-CoV-2 spike D614G protein and then detected by streptavidin-HRP.

Supplementary Figure 3 Analysis of Pearson’s correlation between age and vaccine response in healthy controls and breast cancer patients.
