# Supplementary figures and images for: Prospective longitudinal analysis of antibody response after standard and booster doses of SARS-COV2 vaccination in patients with early breast cancer
Source: Front Immunol. 2022 Nov 17;13:1028102. doi: 10.3389/fimmu.2022.1028102 (PMC9712216; doi:10.3389/fimmu.2022.1028102)

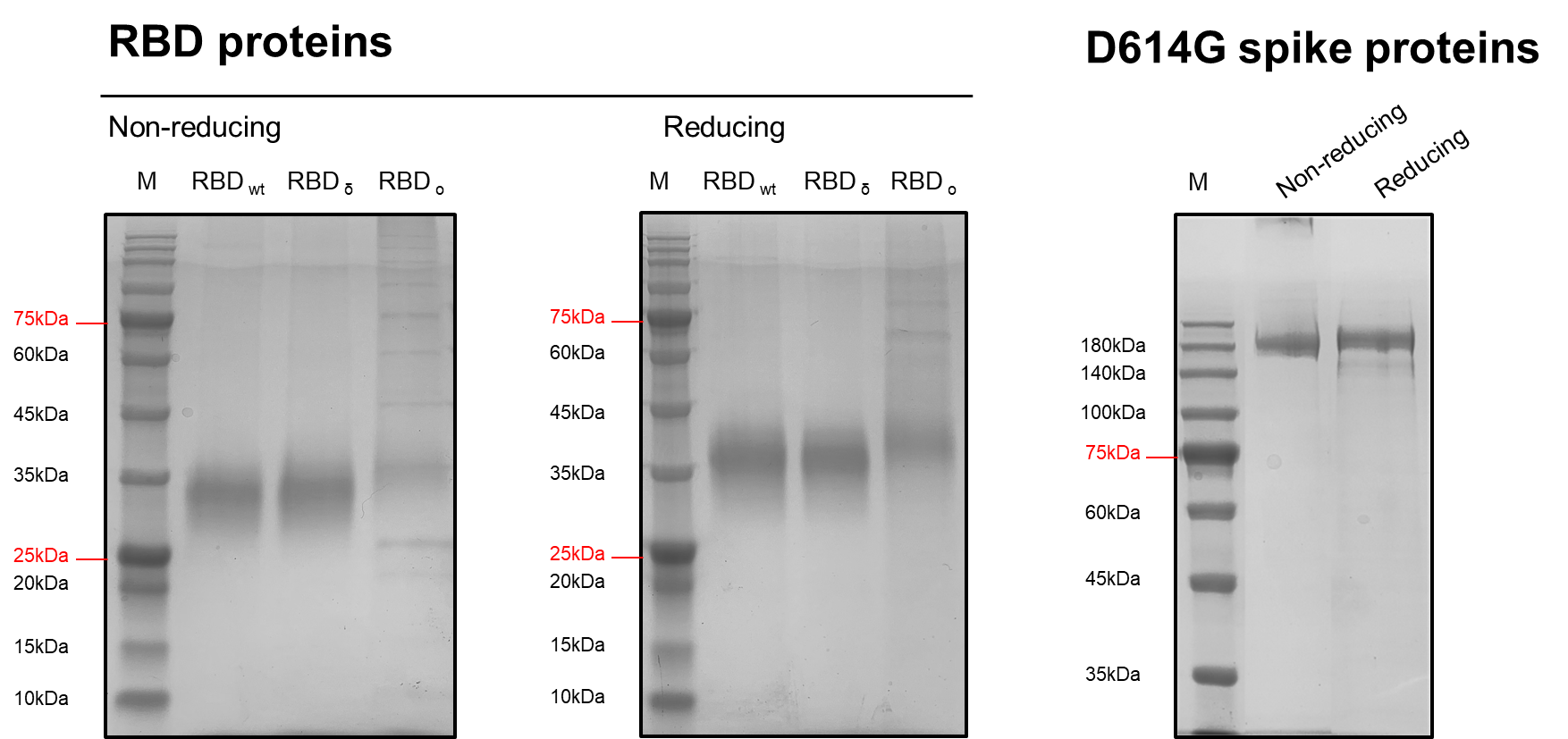

Supplement: Supplementary Figure 1 — Purification of recombinant RBD proteins of SARS-CoV-2 variants and D614G spike protein. The purity of the purified recombinant protein was determined by non-reducing and reducing SDS-PAGE gels. M, protein ladder. [file Image_1.tif]

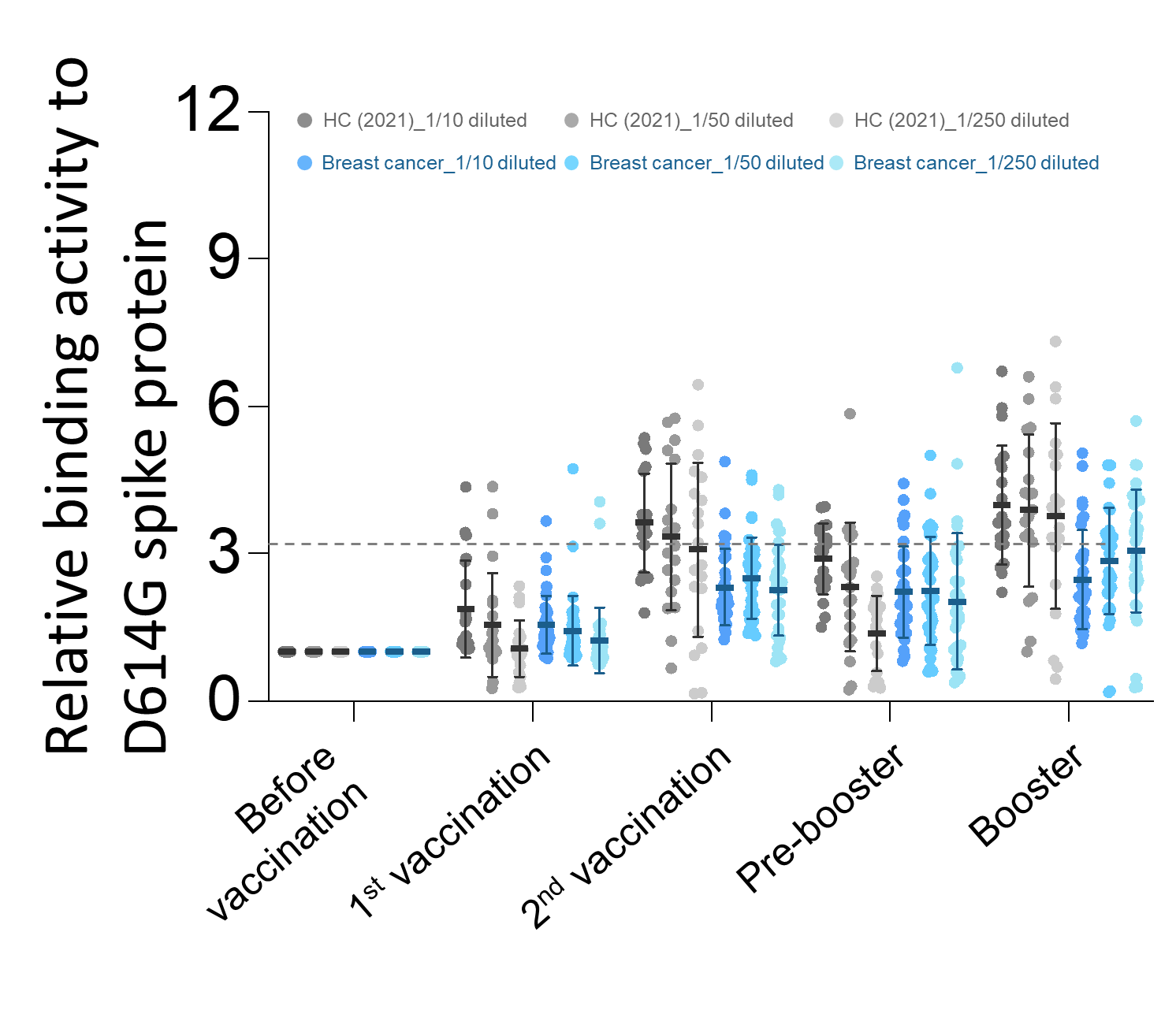

Supplement: Supplementary Figure 2 — Longitudinal humoral immune responses to SARS-CoV-2 spike D614G protein in breast cancer patients. The serially diluted serum IgG binding activities to SARS-CoV-2 spike D614G in the healthy control group and breast cancer patients group were determined using anti-IgG antibodies. The diluted serum samples were incubated with SARS-CoV-2 spike D614G protein and then detected by streptavidin-HRP. [file Image_2.tif]

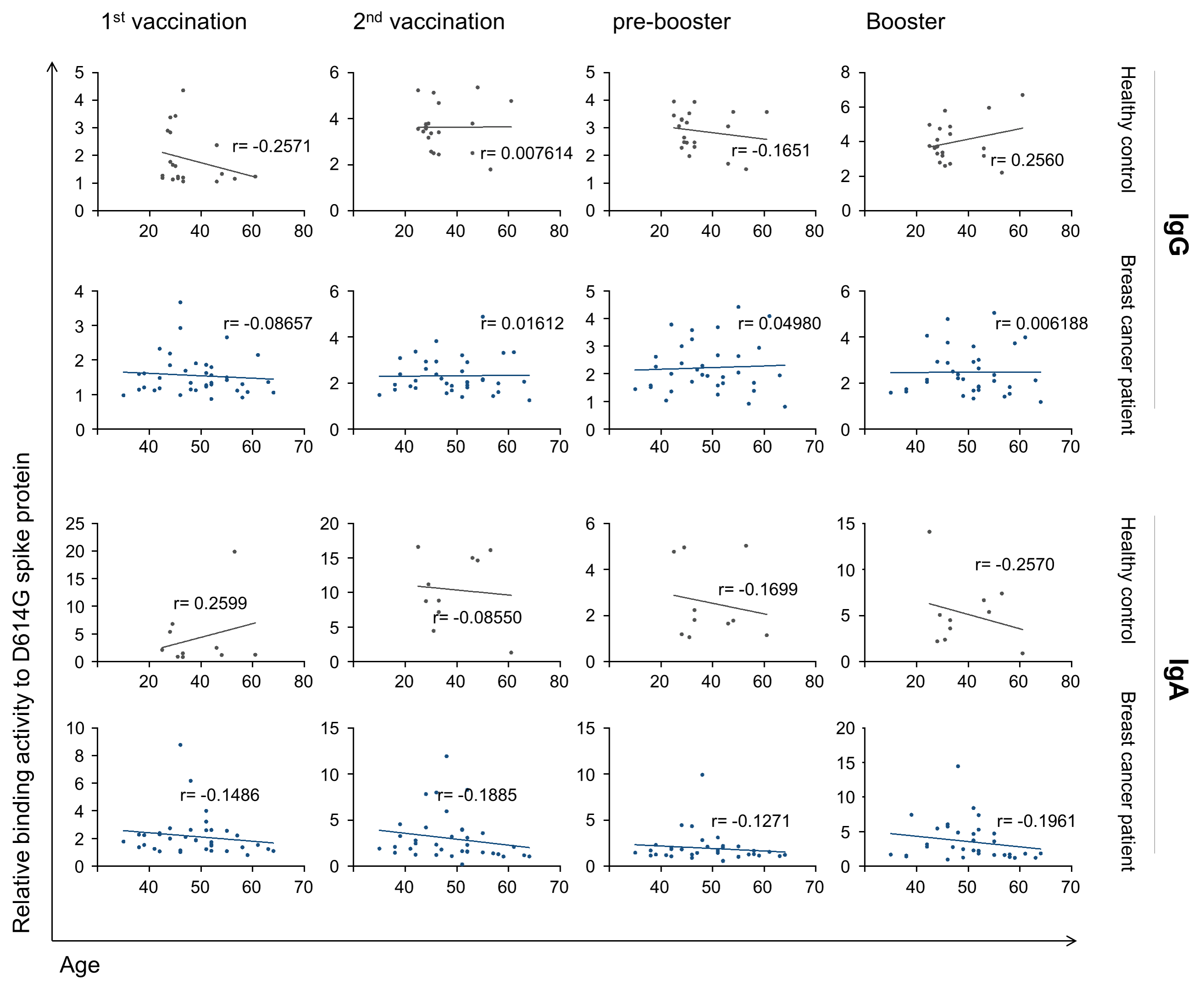

Supplement: Supplementary Figure 3 — Analysis of Pearson’s correlation between age and vaccine response in healthy controls and breast cancer patients. [file Image_3.tif]
